# Exploring the Role of Cognition in the Annual Fall Migration of the Monarch Butterfly (*Danaus plexippus*)

**DOI:** 10.3390/insects12080760

**Published:** 2021-08-23

**Authors:** Robert J. Gegear

**Affiliations:** Department of Biology, University of Massachusetts, Dartmouth, MA 02747-2300, USA; rgegear@umassd.edu

**Keywords:** monarch butterfly, long-term memory, learning ability, cognition, migration, visual system, olfactory system

## Abstract

**Simple Summary:**

Each year, millions of monarch butterflies in eastern North America undergo a spectacular fall migration to overwintering sites in central Mexico, where they remain until returning northward in the spring. In addition to the navigational challenges faced during the southward flight, migratory individuals are also challenged with the foraging task of locating high-quality nectar sources for overwinter survival in the face of unfamiliar floral landscapes that change in complex and unpredictable ways. In the research reported here, a proboscis extension paradigm is used to investigate learning and long-term memory abilities that might help fall migrants meet these unique foraging demands. Male and female migratory and captive-reared individuals were consecutively trained to perform color and odor cue discriminations and then tested for their ability to simultaneously retain reward information associated with each cue in memory without reinforcement over a period of 7 days. Results showed that male and female fall migrants can learn the reward properties of color and odor cues with over 75% accuracy after less than 40 s of exposure and can simultaneously retain visual and olfactory information predicting the availability of floral rewards in memory without reinforcement for at least 7 days. Captive-reared male butterflies also showed the ability to retain visual and olfactory information in long-term memory for 7 days; however, 80% of captive-reared females could not retain color cues in long-term memory for more than 24 h. These novel findings are consistent with the view that monarch butterflies have enhancements to long-term memory that enable them to minimize the amount of time and energy wasted searching for suitable nectar sources during their annual fall migration, thereby optimizing migratory performance and increasing the chance of overwinter survival. The possibility that female monarchs undergo a seasonal change in visual long-term memory warrants further empirical investigation.

**Abstract:**

Each fall, monarch butterflies in eastern North America undergo an extraordinary long-distance migration to wintering areas in central Mexico, where they remain until returning northward in the spring. Migrants survive the overwintering period by metabolizing lipid reserves accumulated exclusively though floral nectar; however, there is little known about how individuals maximize foraging efficiency in the face of floral environments that constantly change in complex and unpredictable ways along their migratory route. Here, a proboscis extension paradigm is used to investigate the role of cognition during the foraging phase of monarch migration. Male and female migratory butterflies were consecutively trained to discriminate between two color and odor cues and then tested for their ability to simultaneously retain the information on the reward value of each cue in memory without reinforcement over a period of 7 days. To gain further insight into cognitive abilities of monarchs as a migratory species, a second set of captive-reared males and females were tested under harnessed conditions at the same time as wild-caught fall migrants. Results showed that male and female migrants can learn the reward properties of color and odor cues with over 75% accuracy after less than 40 s of exposure and can simultaneously retain visual and olfactory information predicting the availability of floral rewards in memory without reinforcement for at least 7 days. Captive-reared male butterflies also showed the ability to retain visual and olfactory information in long-term memory for 7 days; however, 80% of captive-reared females could not retain color cues in long-term memory for more than 24 h. These novel findings are consistent with the view that monarch butterflies, as a migratory species, have enhancements to long-term memory that enable them to minimize the amount of time and energy wasted searching for suitable nectar sources during their annual fall migration, thereby optimizing migratory performance and increasing the chance of overwinter survival. The possibility that female monarchs undergo a seasonal change in visual long-term memory warrants further empirical investigation.

## 1. Introduction

Each fall, monarch butterfly (*Danaus plexippus*) populations in eastern North America undergo a spectacular long-distance migration to overwintering sites in central Mexico, where they remain until remigrating northward in the spring [1,2]. Individuals from the migratory generation show marked differences in physiology and behavior compared to individuals from preceding non-migratory generations, including an extended lifespan, the propensity to congregate with conspecifics, the suppression of all reproductive functions, and the strong drive to fly in a southerly direction [1,2,3,4,5]. Fall migrants also have greater fat reserves than summer butterflies, which they metabolize to maintain themselves over the 5 month wintering period [6]. Fat reserves are accumulated exclusively through the collection of floral nectar along the migratory route [7]. Migrant foraging decisions therefore have important implications for the success of the migratory generation; yet there is surprisingly little known about how fall migrants locate good nectar sources as they fly along their migratory route. Given that floral resource environments constantly change in unpredictable ways, cognition (herein defined as the acquisition, storage, and processing of sensory information [8]) is likely to play a particularly important role in migrant foraging decisions. While cognition in monarchs has been previously studied to a limited extent [9,10,11], the cognitive abilities of migratory monarchs have not been investigated in any detail.

Many animals show special cognitive abilities as an adaptation to meet specific physiological and ecological demands [12,13,14,15,16,17,18], including the unique demands of a migratory way of life [19,20]. In birds, for example, migratory species have been shown to have better memory performance on spatial associative learning tasks than resident species coinciding with the demand in migrants to relocate high quality stopover sites during subsequent migratory seasons [21]. As with birds, migratory monarchs face a dramatically different set of environmental challenges compared to non-migratory butterfly species. Migrants must repeatedly forage in unfamiliar floral environments for long time periods and may travel for days without foraging [22]. Once a floral environment is encountered, for each floral landscape, individuals must decide on whether to stop and forage for nectar or continue flying southward. Once the decision to forage is made, individuals must maximize nectar intake rates over short periods of time under highly complex floral conditions, encountering as many as nine potential nectar sources at a single stopover location [5].

In contrast, butterflies and other flower visitors from non-migratory species continuously forage in familiar habitats [23,24,25], where individuals must acquire detailed knowledge about available nectar sources (plant species) and then constantly monitor how the quality of those familiar resources change over time, with unfamiliar nectar sources being introduced only as new plant species come into bloom. Because of these differences, it is possible that monarchs and non-migratory insect species have evolved specific cognitive abilities that help them to optimize the use of information about their unique foraging environment. A critical first step in testing this possibility is to quantify the cognitive abilities of migratory monarchs in a way that would allow for meaningful comparisons with previous and future reports of cognitive abilities in non-migratory species.

In the present work, a proboscis extension paradigm is used to assess the ability of fall migrants to rapidly form long-lasting memories of floral characteristics predicting the availability of sugar (nectar) rewards. Migrants would benefit from enhancements to memory retention as it would increase foraging efficiency by minimizing the time and energy costs associated with re-learning the nectar properties of previously encountered flowers and with searching in floral habitats previously determined to be unsuitable. In addition, an enhanced ability to reference sensory information gathered in the distant past would improve migratory performance by informing decisions on where to roost and when to switch from flight to foraging along their southward path. Given that flowers transmit sensory information in multiple modalities (e.g., visual and olfactory) and dimensions within each modality (e.g., color and shape within the visual modality), migrants would further benefit from the ability to use sensory information from multiple modalities to make foraging decisions in unfamiliar floral environments. The ability to simultaneously utilize floral information across sensory modalities has been well documented in social bees [26,27] and some lepidopterans [28,29] but has not been investigated in monarchs.

Using a standardized experimental protocol, harnessed male and female fall migrants were trained to discriminate between two colors and odors on consecutive days and were then tested for their ability to recall reward information of stimulus pairs from each modality without reinforcement at 24 h intervals post-training over a 7 day period. Because all previous tests of learning and memory abilities in monarchs have been conducted on free-flying individuals that were reared in captivity, [9,11,30], captive-reared male and female offspring of wild-caught summer butterflies were also trained and tested at the same time as fall migrants. Collectively, the data on foraging-experienced migratory and flower-naïve captive-reared individuals are intended to provide a baseline for more in-depth studies of cognitive abilities in monarchs as a migratory species, thereby facilitating future comparisons with non-migratory butterfly species using the standardized proboscis extension protocol described here. Learning and memory performances of migratory and captive-reared individuals on each sensory task were used to address the following: (1) How long can visual and olfactory information be simultaneously retained in memory? (2) Do males and females differ in their cognitive abilities? (3) Does sensory modality influence learning rate and memory retention?

## 2. Materials and Methods

### 2.1. Butterflies

Captive-reared monarchs were supplied as pupae from late August to early October by Dr. O.R. Taylor at Monarch Watch (University of Kansas, Lawrence, KS, USA). Pupae were offspring of first- and second-generation captive-bred parents. Upon arrival, pupae were suspended in a 24 × 24 × 56″ plastic mesh cage (Bioquip, Rancho Dominguez, CA, USA) placed inside a Percival incubator and housed under summer-like conditions consisting of a 16 h light: 8 h dark light cycle, a constant temperature of 21 °C, and a relative humidity of 70%. These rearing conditions have been shown previously to produce healthy, reproductive, non-migratory individuals [31]. To determine reproductive status prior to testing, 10 females were dissected and checked for the presence of mature oocytes. All females were found to have mature oocytes, indicating that captive-reared individuals were reproductively active.

**Figure 1:**
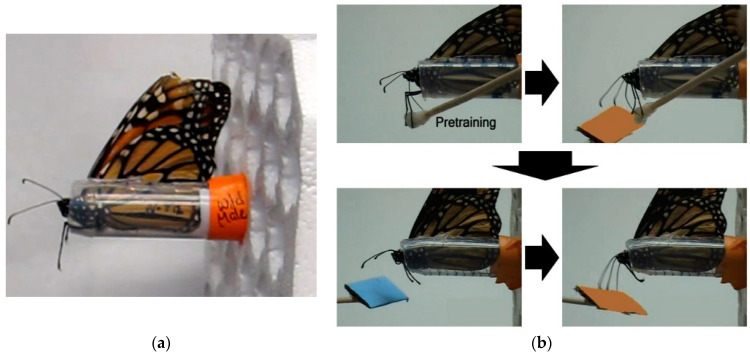
Proboscis extension paradigm for assessing learning and long-term memory in monarch butterflies. (**a**) Individuals were immobilized in an open-ended tubular harness so that only the head, antenna and middle legs were free to move. (**b**; **upper left** panel) A proboscis extension response in monarchs was be elicited by contacting the middle legs with a cotton swab soaked in 20% sucrose solution. All individuals were checked for an intact proboscis extension response prior to experiments (pre-training). (**b**; **upper right** panel) Individuals were separately trained to discriminate between two colors (shown) and odors by reinforcing one stimulus with sucrose reward but not the other. (**b**; **lower** panels) Memory retention was assessed by presenting the unrewarded stimulus for 5 s followed by the rewarded stimulus for 5 s without reinforcement at 24 h intervals over a period of 7 days. For olfactory conditioning, odorants were presented to individuals by depositing them the surface of white foam with the same dimensions (2 cm × 3 cm) as orange and blue foam used for the color discrimination task. See text for more details.

Adults fed on 20% sucrose solution ad libitum from a feeder placed at the bottom of the cage. One week post-emergence, butterflies were individually transferred to a plastic harness (see Figure 1a). The harness consisted of a 15 mL polypropylene conical tube with the bottom 0.5 cm removed and a slot cut down the side so that the head, antenna, and middle legs of the butterfly could be extended while immobilizing the rest of the body. A piece of labelling tape was placed over the open portion of the slot behind the wings of the butterfly to prevent it from sliding out the harness. The harness containing the butterfly was then embedded in a large block of plastic foam such that the legs of the butterfly were suspended in the air. Each block held 16 butterflies. All harnessed butterflies were kept in the incubator under summer-like conditions when not being tested. While in the harness, butterflies were hand fed daily by unrolling their proboscis with a dissecting pin and placing it in a small clear plastic odorless feeder containing 10 µL of 20% sucrose solution. All individuals were allowed to feed *ad libitum*.

Migratory monarch butterflies were captured from September to October while foraging at a stopover site in an open field near Greenfield, MA, USA (latitude 42°59′ N, longitude 72°60′ W). Individuals were placed in glassine envelopes and housed indoors in a separate Percival incubator that had its lighting regime set to fall-like conditions consisting of a 12 h light: 12 h dark lighting regime, a cycled temperature (23 °C during light and 12 °C during dark), and a relative humidity of 70%. The light cycle started at the same time (6 a.m. EST) for both captive-reared and migrant butterflies so that individuals could be tested at the same time of day and during daylight hours. Individuals were hand fed 20% sucrose solution ad libitum once a day for two days and then placed in the harness apparatus as described for captive-reared butterflies. The reproductive status of 10 migratory butterflies was evaluated before, and another subset of 10 after, experiments by checking for the presence of mature oocytes in females. In all cases, females had no mature oocytes, confirming individuals remained in reproductive diapause over the testing period.

### 2.2. Experimental Procedure

Harnessed individuals were checked for an intact proboscis extension response (PER) by contacting their middle legs with a with a cotton-tipped applicator soaked in 20% sucrose solution (wt/wt; Figure 1b, upper left). A small fraction of captive-reared (2/27) and migrant (1/27) individuals did not show a full proboscis extension in response to sucrose application and were excluded from the experiment. Individuals were consecutively trained on a visual and an olfactory cue discrimination task using a differential conditioning procedure [32].

Task training: Individuals were first trained to discriminate between either two color or two odor cues by pairing one stimulus with 20% sucrose solution (rewarded trial) and pairing the other stimulus with distilled water (unrewarded trial). Blue and orange stimuli were used for the visual task and lavender and rose scents were used for the olfactory task. Stimuli were presented at the end of a 15 cm wooden stick approximated 2 cm from the head. For color, a rectangular (2 cm × 3 cm) piece of either orange or blue Creatology^TM^ foam (Michaels Stores, Inc., Irving, TX, USA) was fixed to the end of the stick. For odor, 5 µL of either lavender or rose oil diluted in pentane (1:100) was deposited on white-colored foam of the same size. Prior to experiments, all individuals were tested for a spontaneous response to all color and odor stimuli.

For rewarded trials, individuals were presented with one sensory cue for 10 s. No individuals showed a spontaneous response upon initial presentation of any of the test color and odor cues. Five seconds after introduction of the sensory cue, the middle legs were contacted with the cotton applicator soaked with sucrose solution, resulting in full proboscis extension and subsequent feeding (Figure 1b, upper right). Individuals fed for 5 s and then the cue was removed. Individuals continued feeding for an additional 5 s and then the cotton applicator was removed. The same procedure was used for unrewarded trials with the second sensory cue except that the cotton applicator was soaked with distilled water, which did not induce proboscis extension and feeding. The order and number of rewarded (A) and unrewarded (B) trials, which were the same for all individuals were: A-A-B-B-A-B-A-A-B-A-B-B-A-B-A with an intertrial interval of 10 min.

The day following discrimination training in the first modality (approximately 24 h), individuals went through the same training procedure with the stimulus pair from the second modality (Day 2 of training). To control for potential order effects, the rewarded stimulus and sensory modality trained on Day 1 and Day 2 was balanced among individuals. Responses of individuals to presentation of test stimuli prior to sucrose or water delivery was scored as follows: a full extension of the proboscis was given a value of 1 and no extension of the proboscis was assigned a value of 0, yielding a binary dataset.

A total of 25 captive-reared (14 M, 11 F) and 26 migratory (13 M, 13 F) butterflies were consecutively trained in color and odor discrimination tasks. Task performance was expressed as a learning score, which was calculated by subtracting the total positive responses to the unrewarded stimulus from total positive responses to the rewarded stimulus over the last 8 learning trials (4 rewarded and 4 unrewarded). Learning scores ranged from 0 (no learning) to 4 (maximum learning performance).

Long-term memory testing: Only individuals with a learning score of 1 or greater, indicating at least 25% stimulus discrimination accuracy, were subsequently tested for memory retention, resulting in 22 captive-reared (13 M, 9 F) and 23 migrant (11 M, 12 F) butterflies. Memory trials started approximately 24 h after the last stimulus conditioning trial of the learning experiment for a given modality and were repeated every 24 h over a 7 day period. Note that 24 h memory tests for stimulus pairs used in Day 1 training were conducted after Day 2 stimulus training. For each trial, individuals were first presented with the unrewarded stimulus alone for 5 s and then immediately presented with the rewarded stimulus alone for 5 s (Figure 1b, lower panels). As with task training, a full extension of the proboscis in response to presentation of either test stimulus was assigned a value of 1, and no extension in response to a stimulus was assigned a value of 0. All test stimuli were unreinforced (no delivery of 20% sucrose or water) during memory testing, but individuals were hand fed to satiation 30 min after each memory trial to keep them in good condition throughout the 7 day memory testing period. For each sensory modality, a long-term memory score was calculated by subtracting the total positive responses to the previously unrewarded stimulus from total positive responses to the previously rewarded stimulus over the 7 memory trials. Long-term memory scores ranged from 0 (no memory retention) to 7 (complete memory retention of all stimulus reward properties).

Data analysis: A two-way repeated-measures ANOVA was performed in R [33] with nlme [34] to compare the main effects of sex and stimulus modality and interactions between sex and stimulus modality on learning and long-term memory scores. Separate analyses were run for captive-reared and migratory butterflies. Pairwise comparisons were performed using the emmeans package [35].

## 3. Results

**Figure 2:**
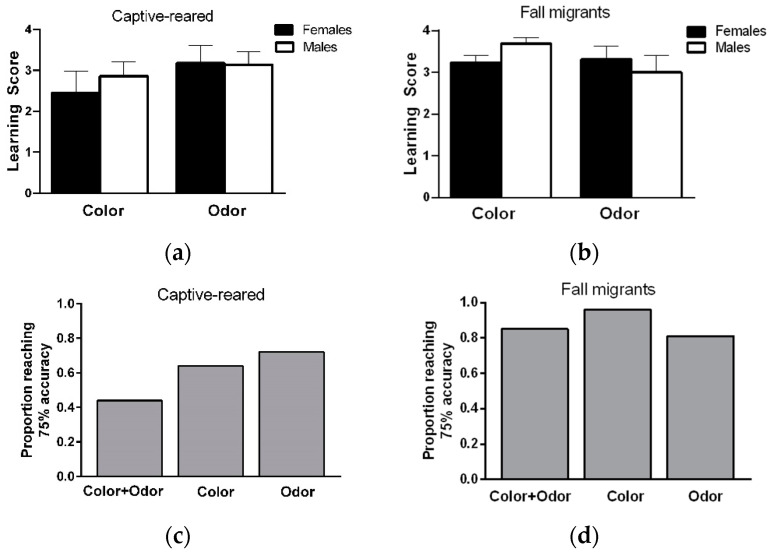
Learning ability of captive-reared and migratory monarch butterflies. (**a**) Mean (+/−) learning scores for male and female captive-reared and (**b**) fall migrants on color and odor discrimination tasks. Learning scores were calculated by subtracting the total proboscis extension responses to the unrewarded stimulus from the number of responses to eight training trials (four rewarded and four unrewarded). A learning score of 4 represents maximum discrimination learning performance. (**c**,**d**) The proportion of individuals achieving 75–100% accuracy on color and odor discrimination tasks. ‘Color + Odor’ shows the proportion of individuals reaching 75% accuracy on color and odor tasks. *n* = 25 for captive-reared butterflies (14 M, 11 F) and 26 for fall migrants (13 M, 13 F).

Learning ability: Learning scores did not differ between stimulus pairs within each modality for captive-reared (orange versus blue; females: *t* = 0.06, *df* = 9, *p* = 0.95; males: *t* = 0.73, *df* = 12, *p* = 0.48; rose versus lavender; females: *t* = 0.45, *df* = 9, *p* = 0.66; males: *t* = 0.85, *df* = 12, *p* = 0.41) and migratory (orange versus blue; females: *t* = 0.073, *df* = 11, *p* = 0.94; males: *t* = 0.98, *df* = 11, *p* = 0.35; rose versus lavender; females: *t* = 1.39, *df* = 11, *p* = 0.19; males: *t* = 0.0, *df* = 11, *p* = 0.99) butterflies. The were no significant effects of sex (F_1,23_ = 0.20, *p* = 0.65), sensory modality (F_1,23_ = 1.44, *p* = 0.24), and sex × sensory modality interaction (F_1,23_ = 0.3, *p* = 0.59) on the learning scores of captive-reared butterflies (Figure 2a). Similarly, there were no significant effects of sex (F_1,24_ = 0.071, *p* = 0.79), sensory modality (F_1,24_ =1.18, *p* = 0.29, and sex × sensory modality interaction (F_1,24_ = 1.85, *p* = 0.18) on the learning scores of fall migrants (Figure 2b).

When considering learning performance on color and odor discrimination tasks together, a total of 22/25 captive-reared (9/11 F, 13/14 M) and 23/26 migrants (12/13 F, 11/13 M) showed a learning score of 3 or greater (at least 75% accuracy) on both tasks, indicating that a high proportion of individuals could learn and remember information from two sensory modalities at the same over short time periods. Most male and female captive-reared butterflies showed a high level of learning on the color or odor discrimination task (Figure 2b), but only 44% of individuals showed high learning performance on color and odor tasks (Figure 2c). In contrast, almost all male and female migrants (over 80%) showed high levels of learning performance on color and odor tasks (Figure 2d).

**Figure 3:**
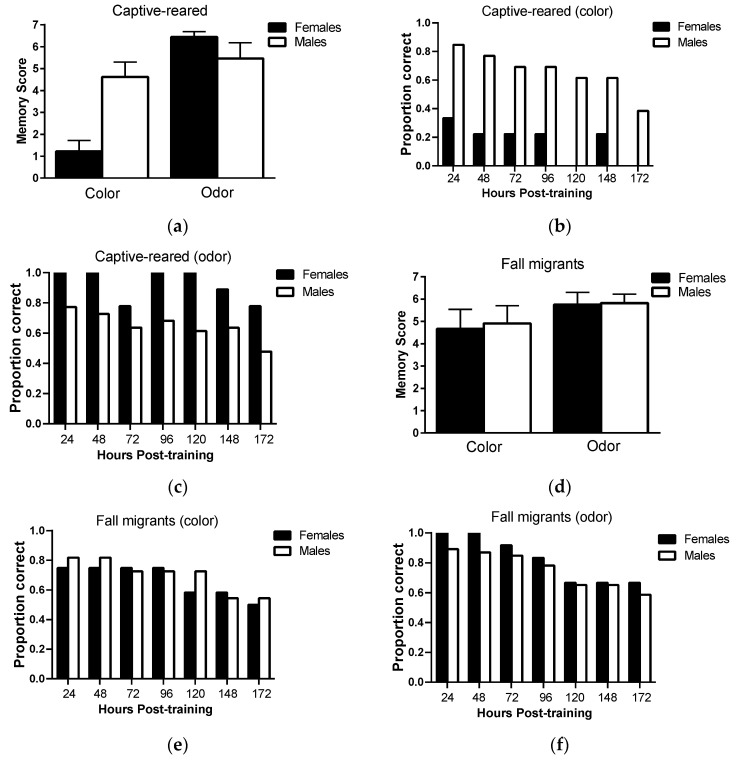
Long-term memory retention of visual and olfactory information in captive-reared and migratory monarch butterflies. (**a**) Mean (+/−SE) long-term memory scores for male and female captive-reared and (**b**) migratory monarchs presented on color and odor discrimination tasks. Memory scores were calculated by subtracting the total number of proboscis extension responses to the previously unrewarded sensory cue from the total number of responses to the previously rewarded sensory cue without reinforcement over the seven memory trials. A memory score of 7 indicates 100% recall accuracy over the 1 week testing period. (**c**,**d**) The proportion of male and female captive-reared and (**e**,**f**) migratory butterflies retaining the correct reward properties of color and odor cues at each memory trial (hour post-training). Letters denote the results of pairwise comparisons at *p* < 0.01. See text for details. *n* = 22 for captive-reared butterflies (13 M, 9 F) and 23 for fall migrants (11 M, 12 F).

Long-term memory retention: There was no significant main effect of sex (F_1,20_ = 3.37793, *p* = 0.08) on long-term memory scores in captive-reared butterflies (Figure 3a; however, there was a significant main effect of sensory modality (F_1,20_ = 18.1, *p* = 0.0004) and a significant sex × sensory modality interaction (F_1,20_ = 12.0, *p* = 0.002). Pairwise comparisons revealed that females had significant lower long-term memory scores than males on the color task (*p* = 0.001) but not the odor task (*p* = 0.29). Females also had lower long-term memory scores on the color task than the odor task (*p* < 0.0001) but no such differences were observed in males (*p* = 0.31). Only 30% of female captive-reared butterflies retained color information 24 h post-training and no females retained color information 172 h post-training compared to 80% and 38% of captive-reared males (Figure 3b, respectively. However, captive-reared females showed a high capacity for retaining olfactory information, with 100% of individuals correctly responding to odor cues 120 h post-training and 78% responding correctly at 172 h post-training (Figure 3c). Captive-reared males showed similar levels of memory retention for visual and olfactory information.

In fall migrants, there was no significant main effect of sex (F_1,21_ = 0.05, *p* = 0.83) and sensory modality (F_1,21_ = 2.19, *p* = 0.15) and no significant sex × sensory modality interaction (F_1,21_ = 0.02, *p* = 0.89) on long-term memory scores (Figure 3d). Female and male fall migrants showed consistently high levels of memory performance on both color and odor tasks over the testing period (Figure 3e,f). Over 70% of individuals responded correctly to color and odor cues at 96 h post-testing, falling to 50% and 60% correct responses to color and odor cues, respectively, at 172 h post-training.

## 4. Discussion

The results of this study provide the first evidence for exceptional learning and long-term memory abilities in foraging-experienced fall migrant and experience-naïve captive-reared monarch butterflies. Fall migrants demonstrated the capacity to quickly learn color– and odor–reward associations in succession with a high level of proficiency, reaching 80% accuracy levels on both color and odor tasks with less than 1 min of exposure to stimulus pairs. On memory tests, over 50% of migratory males and females were able to remember color and odor reward properties without reinforcement for at least 7 days, indicating that migrants can simultaneously retain stimuli from multiple sensory modalities in long-term memory over substantial time periods. Captive-reared male monarchs without any previous experience foraging on flowers also showed a high degree of long-term memory retention on color and odor tasks, with 40% of individuals correctly recalling visual and olfactory cues after 7 days without reinforcement. This is the longest, to my knowledge, reported retention period for visual and olfactory information simultaneously encoded in memory for any insect. The learning rates and memory retention intervals for male and female migrants reported here are also dramatically higher than those reported in previous studies of free-flying monarchs reared in captivity [9,11,30]; however, further direct comparisons of cognitive abilities between wild-caught migratory and non-migratory monarchs with the same level of foraging experience are needed to determine the nature of these differences.

It has been assumed that color learning and memory in fall monarchs is strongly limited by innate preferences for certain colors, specifically the colors orange and yellow [11]; however, migrants and captive-reared individuals did not show a learning or memory bias for orange versus blue color cues, providing little support for this assumption. Moreover, fall migrants and captive-reared males did not show any differences in cognitive performance on color and odor tasks, indicating that they are equally proficient at acquiring visual and olfactory information from the environment and at maintaining visual and olfactory cues predicting reward availability in long-term memory at the same time. Collectively, these results suggest that cognition plays a more prominent role in foraging decisions of monarchs during the fall migration than innate preference.

The present findings are consistent with the view that monarchs, as a migratory species, have enhanced long-term memory across sensory modalities to better locate good nectar sources in unfamiliar floral landscapes more rapidly and efficiently, thereby optimizing migratory performance and increasing the probability of survival during the overwinter period. Such enhancements could arise through foraging experiences during migration, seasonal changes in cognitive ability, or increased selection pressure for superior cognitive abilities needed for successful overwintering [21]. Although the design of the present study does not allow for definitive separation of these possibilities in monarchs, the fact that captive-reared males showed the same high level of visual and olfactory memory retention as wild-caught fall migrants suggests that monarchs as a species have enhanced long-term memory abilities. It is also interesting to note that captive-reared female monarchs without any previous floral experiences, despite having substantially limited long-term visual memory, had exceptional olfactory long-term memory ability, paralleling those found for fall migrants and captive-reared males. This suggests that females may undergo a seasonal change in the ability to retain visual information in memory. In the future, the same proboscis extension protocol will be used to explore these possibilities by directly comparing cognitive performance of migratory monarchs with wild-caught non-migratory monarchs and with individuals from closely related non-migratory species with and without foraging experience, as has been done for migratory bird species [20,21].

This study is also the first to show that visual long-term memory capacity differs between non-migratory monarch males and females. Specifically, only 30% of captive-reared females retained color information in memory at 24 h post-training and no females retained color information in memory at 7 days post-training, compared to 85% and 38% of captive-reared males, respectively. Captive-reared male and female monarchs did not, however, differ in color learning ability, indicating that cognitive differences were limited to memory. Somewhat surprisingly, male and female monarchs reared indoors did not show similar differences in olfactory long-term memory capacity. In fact, females consistently outperformed males across odor memory trials, indicating the cognitive deficits in females were sensory modality specific. One possible explanation for the reduced color memory performance of females is that they weigh visual and olfactory information differently in memory and therefore preferentially ‘discard’ visual information from memory when olfactory information is also available. Such prioritization of sensory cues has been observed in other lepidopterans, including *Danaus* species [26,27]. For example, *Danaus genutia* strongly prefers odor over color cues, while *D. chrysippus* strongly prefers the reverse. It is also possible that captive-reared females are simply unable to retain color information in long-term memory.

It is not clear why visual long-term memory of reproductively active monarchs might be greater in males than females. Sex differences in cognitive ability observed in birds and small mammals have been attributed to an increase in the cognitive demands of one sex [36,37]. In flower-visiting insects, cognitive enhancements in females have been proposed the help them collect more nectar to meet the increased energetic demands of egg production and oviposition [38,39,40]. Although reproductively active female monarchs would have increased nectar demands for similar reasons, monarch mating is unusual in that males force females to copulate using a highly physical “take down” strategy instead of trying to attract them through chemical attractants or elaborate aerial displays [41,42]. Males are known to travel at higher flight speeds than females and over greater distances [43]. It is therefore possible that monarch males have greater cognitive abilities than females because they have greater nectar requirements when reproductively active, a sex difference that would not be present in migratory individuals. While males would certainly benefit from cognitive functions that increase nectar intake to meet such demands, the foraging benefits of specific changes to visual long-term memory are not obvious.

An alternative explanation is that males have enhanced long-term memory to meet the high memory demands of mate search. Males are known to regularly patrol the same area containing wildflowers and host plants for days in search of females [43]. A superior ability to form long-lasting memories of visual features associated with the spatial distribution of host and nectar plants may enable males to search for females more efficiently and over larger areas than males without such abilities, providing them with a competitive advantage. Sex differences in long-term visual memory related to mate search are well known in bird and mammals [44]. Long-term visual memory has also been shown to play a major role in the navigational system of many migratory bird species [20,45], and may play a similar role in the navigational mechanism of monarchs. Further empirical examination of spatial abilities in non-migrant and migratory monarchs would be beneficial and provide further insight into potential cognitive adaptations to a migratory lifestyle in this iconic insect species.

## Data Availability

The data that support the findings of this study are available from the corresponding author, RJG, upon reasonable request.
